# The Implications of an Aging Undocumented Population in Illinois

**DOI:** 10.1093/geroni/igaa057.282

**Published:** 2020-12-16

**Authors:** Padraic Stanley, Brittney Lange-Maia, Raj Shah

**Affiliations:** 1 Rush University Medical Center, Chicago, Illinois, United States; 2 Rush University, Chicago, Illinois, United States

## Abstract

Both academic and public access literature regarding undocumented immigrant older adults is incredibly sparse, and there is no known literature forecasting its growth or the impact of that growth. Therefore, this two-part project commissioned a demographic report to analyze the current undocumented population in Illinois and its projected growth by 2030. We then convened a cross-sector collaborative of leaders in aging, healthcare, immigrant services, and their intersections to discuss the direct practice and structural policy implications of an aging undocumented community. According to this report, the number of undocumented immigrants who are over the age of 55 in Illinois will grow drastically by the year 2030. In fact, the population aged 65 to 74 will increase from 3,392 to 47,271 (more than a twelve-fold increase), and those aged 75-84 will increase from 594 to 7,621 (an eleven-fold increase). Under the current immigration and healthcare systems, without access to Medicaid, Medicare, or any public benefits, these individuals have extremely limited access to services necessary for older adults to age successfully in place—rehab, nursing homes, home health, homemaker services, long-term care, etc. The study also analyzed demographics from the current undocumented older adult population. This poster will summarize the findings of the demographic portion of the report, the themes and findings of the structural and practice implications of an aging undocumented community in Illinois, and provide policy recommendations to prepare for the wave of undocumented older adults by 2030.

